# Supplementary material for: High Surface Reactivity and Biocompatibility of Y2O3 NPs in Human MCF-7 Epithelial and HT-1080 Fibro-Blast Cells
Source: Molecules. 2020 Mar 3;25(5):1137. doi: 10.3390/molecules25051137 (PMC7179248; doi:10.3390/molecules25051137)
Supplement: Supplementary file 1 [file molecules-25-01137-s001.pptx]

## Slide 1
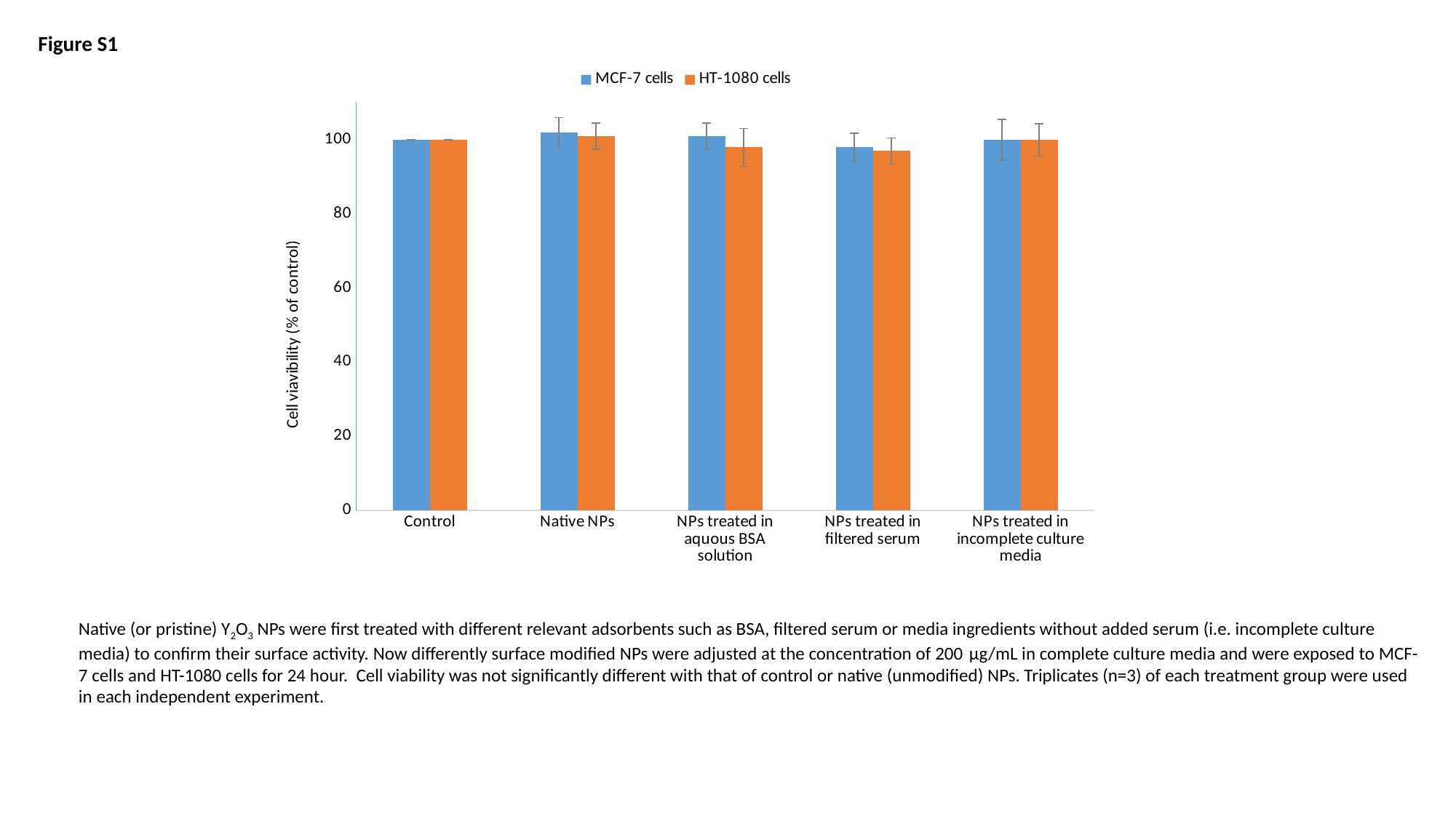

Figure S1
### Chart
| Category | MCF-7 cells | HT-1080 cells |
|---|---|---|
| Control | 100.0 | 100.0 |
| Native NPs | 102.0 | 101.0 |
| NPs treated in aquous BSA solution | 101.0 | 98.0 |
| NPs treated in filtered serum | 98.0 | 97.0 |
| NPs treated in incomplete culture media | 100.0 | 100.0 |Native (or pristine) Y2O3 NPs were first treated with different relevant adsorbents such as BSA, filtered serum or media ingredients without added serum (i.e. incomplete culture media) to confirm their surface activity. Now differently surface modified NPs were adjusted at the concentration of 200 μg/mL in complete culture media and were exposed to MCF-7 cells and HT-1080 cells for 24 hour. Cell viability was not significantly different with that of control or native (unmodified) NPs. Triplicates (n=3) of each treatment group were used in each independent experiment.

## Slide 2
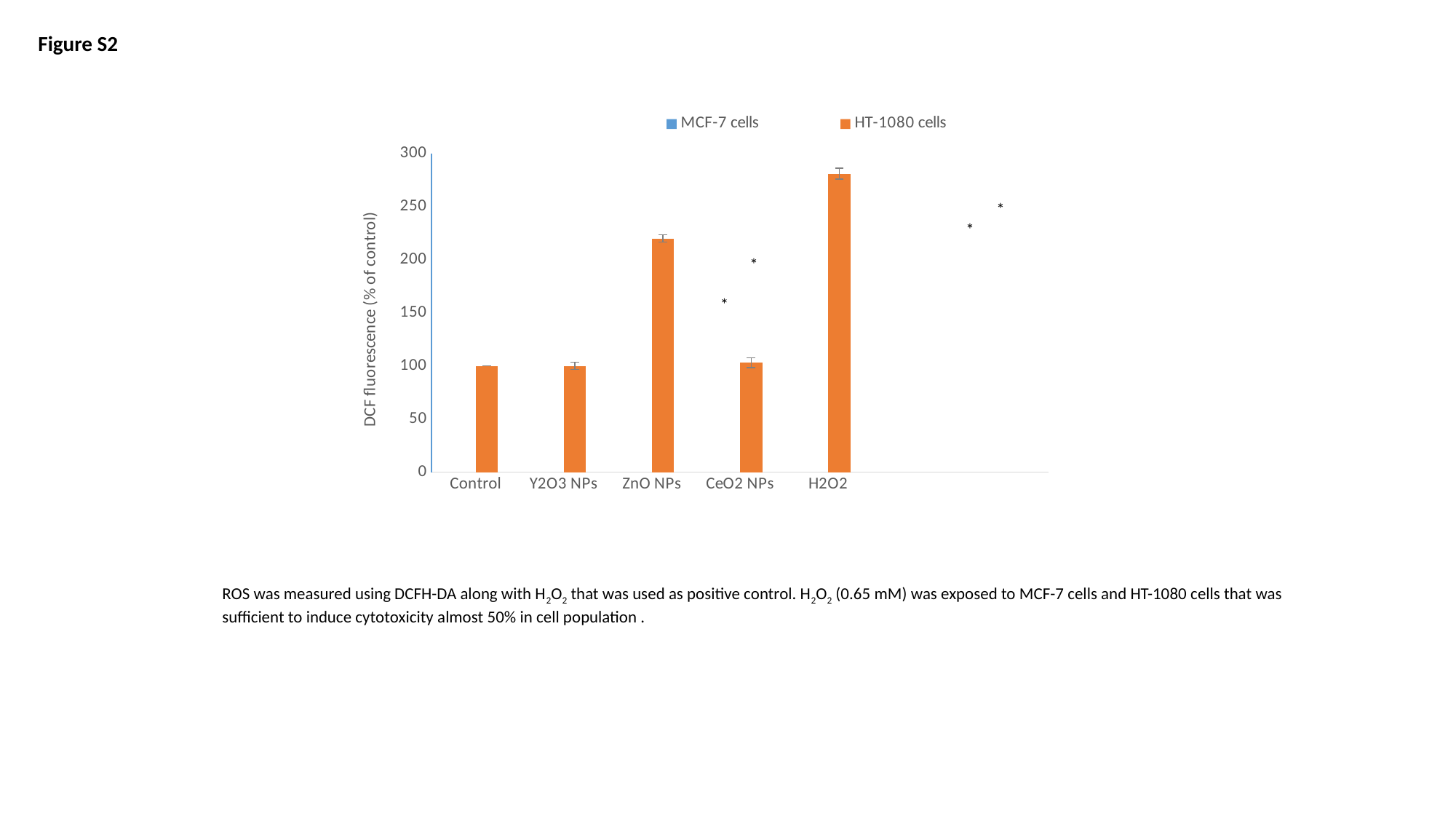

Figure S2
### Chart
| Category | MCF-7 cells | HT-1080 cells |
|---|---|---|
| Control | 100.0 | 100.0 |
| Y2O3 NPs | 103.0 | 100.0 |
| ZnO NPs | 176.0 | 220.0 |
| CeO2 NPs | 101.0 | 103.0 |
| H2O2 | 258.0 | 281.0 |*
*
*
*
ROS was measured using DCFH-DA along with H2O2 that was used as positive control. H2O2 (0.65 mM) was exposed to MCF-7 cells and HT-1080 cells that was sufficient to induce cytotoxicity almost 50% in cell population .

## Slide 3
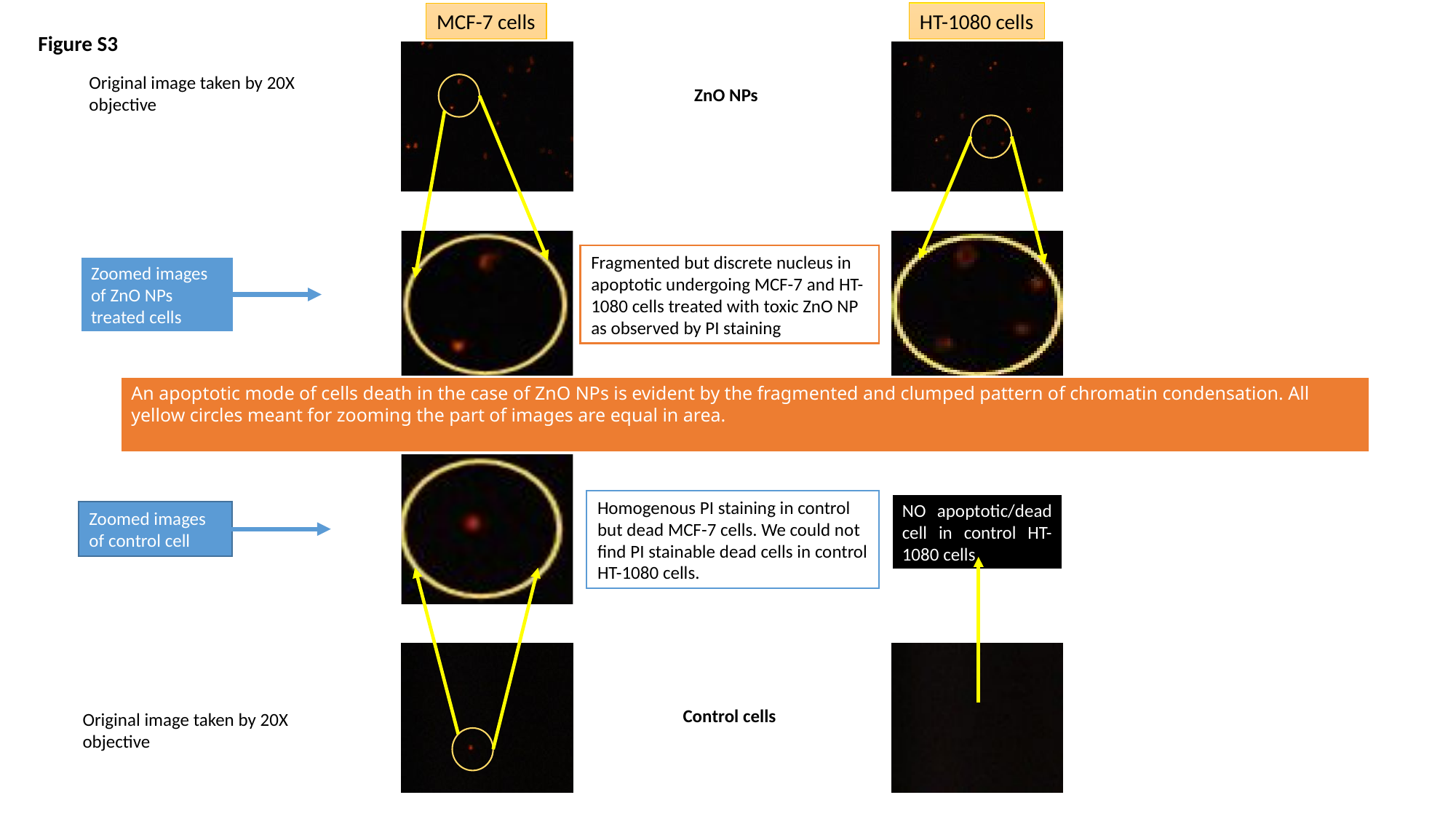

HT-1080 cells
MCF-7 cells
Figure S3
Original image taken by 20X objective
ZnO NPs
Fragmented but discrete nucleus in apoptotic undergoing MCF-7 and HT-1080 cells treated with toxic ZnO NP as observed by PI staining
Zoomed images of ZnO NPs treated cells
An apoptotic mode of cells death in the case of ZnO NPs is evident by the fragmented and clumped pattern of chromatin condensation. All yellow circles meant for zooming the part of images are equal in area.
Homogenous PI staining in control but dead MCF-7 cells. We could not find PI stainable dead cells in control HT-1080 cells.
NO apoptotic/dead cell in control HT-1080 cells
Zoomed images of control cell
Control cells
Original image taken by 20X objective
